# Estimation of Validity of A-Mode Ultrasound for Measurements of Muscle Thickness and Muscle Quality

**DOI:** 10.3390/bioengineering11020149

**Published:** 2024-02-01

**Authors:** Ji-Won Lee, Seung-Ug Hong, Ju-Hee Lee, Sung-Yun Park

**Affiliations:** 1 Department of Korean Medicine, Graduate School, Dongguk University, Goyang 10326, Republic of Korea; behelia11@gmail.com; 2 Department of Ophthalmology, Otolaryngology and Dermatology, College of Korean Medicine, Dongguk University, Goyang 10326, Republic of Korea; heenthsu@duih.org; 3 College of Korean Medicine, Dongguk University, Goyang 10326, Republic of Korea; jh1548@dongguk.ac.kr; 4 Department of Diagnostics, College of Korean Medicine, Dongguk University, Goyang 10326, Republic of Korea

**Keywords:** amplitude modulation, muscle thickness, muscle quality, subcutaneous fat thickness

## Abstract

This study aimed to determine whether amplitude modulation (A-mode) ultrasound (US) provides accurate and reliable measurements comparable to those obtained using brightness modulation (B-mode) US under diverse conditions. Thirty healthy participants (15 women and 15 men) underwent measurements of subcutaneous fat thickness (SFT), muscle thickness (MT), and muscle quality (MQ) in the trapezius and biceps brachii muscles using both US modes before and after exercises designed to stimulate the respective muscles. Among the three key indices, the results demonstrated the high validity of the A-mode, with minimal mean differences (MDs) between the two devices less than 0.91 mm and intra-class correlation coefficients (ICCs) exceeding 0.95 for all measures. In addition, the correlation coefficients between the error scores and average scores for the trapezius and biceps brachii suggested no evidence of systematic error. The trapezius MT and MQ significantly increased, and the biceps brachii MT significantly increased after the exercises (*p* < 0.05). Notably, both the A- and B-modes exhibited the same trend in these post-exercise changes in the muscle. This study suggests that low-cost and low-resolution A-mode US provides measurements of SFT, MT, and MQ similar to the more expensive, high-resolution B-mode imaging. A-mode US is an affordable and portable alternative for muscle assessment.

## 1. Introduction

Muscles, as a key representative of body composition, play a crucial role in both physical performance and metabolic health [1]. Low muscle mass and strength are associated with worsening activities in daily living, which predict clinically relevant adverse events in older populations [2]. Changes in the muscle distribution reflect a risk of musculoskeletal, metabolic, cardiovascular, and cerebrovascular disorders [1,2,3]. Therefore, the continuous observation of muscle changes is crucial for health monitoring, highlighting the need to develop accurate and reliable tools for muscle evaluation.

While magnetic resonance imaging (MRI) and computed tomography (CT) stand as gold standards for minimally invasive muscle mass measurement, the assessment of muscle quality (MQ) remains a point of controversy [4,5]. Ultrasound (US) is rapidly emerging as a muscle evaluation method because of improvements in portability and technology owing to recent software developments. Offering a non-invasive, ionizing-radiation-free alternative, US has proven to be highly versatile, swiftly scanning soft tissues, and excelling in soft tissue imaging [5,6]. In contrast to MRI, which offers a resolution of 1.3–2.0 mm for a whole-body scan, the latest US can provide a resolution of 0.1 mm (18 MHz probe) to give the highest measurement accuracy [7,8]. US allows sonographers to check muscle changes promptly, even at curved sites, by performing quick and dynamic tests [6]. Recently, echo intensity (EI) derived from US images has emerged as an indicator of skeletal MQ and composition [5,9]. Symposium reports have recently highlighted the efficacy of the skeletal muscle EI as an MQ indicator [10].

However, US may be limited to patients with obesity, requiring several probes to measure various depths or resolutions, and it poses diagnostic challenges as the interpretation of the images is user-dependent [6]. Nevertheless, many studies have reported the validity and reliability of US as a muscle evaluation method and have suggested that it is a practical diagnostic tool for viable muscle assessment in communities and hospitals [4,5,6,7]. 

Among the several methods of representing the reflected signal in ultrasonic measurements, the primary techniques are amplitude modulation (A-mode), which reveals changes in amplitude in one dimension, and brightness modulation (B-mode), which presents the A-mode signal as the brightness of dots in 2D images [11]. Considering the value of images, the B-mode is commonly utilized; however, it is costlier than the A-mode and demands expertise, training, and time for result interpretation [11]. This presents a barrier for many independent sonographers aiming to assess muscles in the field or within fitness establishments. Therefore, if A-mode US is sufficiently validated as a suitable method for muscle evaluation through a non-inferiority comparison with measurements obtained by B-mode, A-mode US could be considered a cost-effective alternative across diverse healthcare settings, particularly those with limited resources.

While some studies have compared A-mode and B-mode for the measurement of subcutaneous fat, research on the means to assess MQ remains unpublished [11,12,13]. Therefore, this study aimed to establish the validity of A-mode US as a clinically viable tool for measuring muscle mass and quality. Our focus was on the trapezius and biceps brachii muscles, given their crucial roles in basic muscle US protocols, myopathy screening, and motor neuron disease examinations [4]. Moreover, these muscles are the primary targets of upper-body exercises.

Additionally, we integrated exercise variables into our US measurements to determine whether A-mode US could provide accurate and reliable measurements comparable to those obtained using B-mode US under diverse conditions. Ultimately, this study aimed to compare and analyze the trapezius and biceps brachii muscles before and after exercise by employing both A- and B-mode US, thereby proving the appropriateness of A-mode US as a method for assessing muscles.

The main contributions of our work are as follows:

(1) The development of algorithms for A-mode US to assess muscle thickness (MT) and MQ.
(2) An assessment of the precision of A-mode US in measuring subcutaneous fat thickness (SFT), MT, and MQ by comparing it with B-mode US measurements.
(3) Validation of the accuracy of A-mode US in detecting post-exercise muscle changes similar to B-mode US.
(4) Illustration of the efficacy and practicality of A-mode US as a valuable tool for muscle assessment in healthcare settings with limited resources.

The rest of this paper is structured as follows. In Section 2, we propose an algorithm and conduct experiments to assess the precision of A-mode US in measuring muscles as well as detecting post-exercise muscle changes. In this section, we also statistically analyze the validity of A-mode US by comparing it with B-mode measurements. In Section 3, we present the results of the experiment. In Section 4, we provide a deeper interpretation of the findings, make comparisons with other studies, discuss the limitations of the study, and formulate some suggestions for future research. In Section 5, we wrap up our main conclusions and provide outlooks based on the findings of this paper.

## 2. Materials and Methods

### 2.1. Study Population

Thirty healthy participants aged between 19 and 50 years were enrolled in this study as first-come, first-serve applicants, with no distinction made between the sexes in the order of application. The exclusion criteria included participants with musculoskeletal injuries, those who did not agree to participate in the research, individuals in vulnerable environments (such as Korean medical students, medical students, nursing students, hospital staff, pregnant women, individuals with intellectual disabilities who could not express their will, and people with disabilities). This study was approved by the Institutional Review Board of the Dongguk University Ilsan Oriental Hospital (protocol no. DUIOH 2022-07-006-003). All the participants provided written informed consent to participate in the study.

### 2.2. US Scanning

This study was conducted at the Korean Medicine Ophthalmology, Otolaryngology, and Dermatology Outpatient Clinic of Dongguk University Ilsan Oriental Medicine Hospital in Ilsan. The clinical temperature and humidity were maintained according to the US manufacturer’s guidelines. US measurements were conducted before and after exercise in a single session.

#### 2.2.1. Identification and Marking of the US Measurement Site

**Figure 1:**
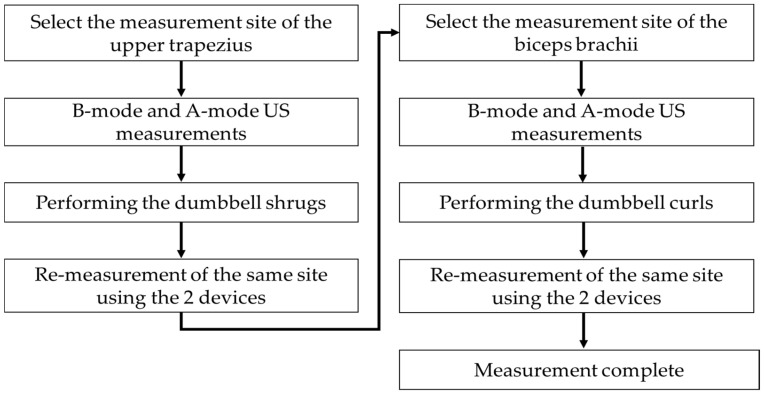
Ultrasound (US) scanning procedures.

The US scanning process is illustrated in Figure 1. The US measurement sites were selected based on previous studies: the midpoint of the upper trapezius and biceps brachii muscles on the participant’s dominant arm [4]. Anatomical marks and US sites were indicated using a hypoallergenic surgical marking pen: the upper trapezius measurement site (midpoint between the acromion process of the scapula and the 7th cervical vertebrae) and the biceps brachii measurement site (midpoint between the acromion process and the elbow crease).

An experienced sonographer with more than 10 years of experience conducted all US measurements on the 30 participants. The upper trapezius muscle strength was measured with the participants instructed to sit and relax with 0° shoulder abduction. Subsequently, the biceps brachii muscle was measured with the participants lying comfortably on a bed in the supine position with their arms in supination.

#### 2.2.2. B-Mode and A-Mode US Evaluations

A sufficient amount of water-soluble gel was applied to the marked sites and US transducer head. Each participant then underwent B-mode US evaluation using a Healcerion SONON 300 L device (Healcerion, Seoul, Republic of Korea) with a linear probe (central frequency of 5 MHz). The US transducer was positioned perpendicular to each muscle at the marked sites. After completing the B-mode US measurement, the center of the B-mode US measurement site was designated as the A-mode US measurement site and marked with a marking pen. The gel was reapplied, and A-mode evaluations were conducted with an XTR-2020 Square/Spike Ultrasonic Pulser/Receiver (MKC Korea, Seoul, Republic of Korea) at a set frequency of 5 MHz. As the A-mode single probe uses one piezoelectric element, the signal waveform changes with minute movements, ensuring that the ultrasonic waves are irradiated as vertically as possible.

#### 2.2.3. Post-Exercise B-Mode and A-Mode US Re-Measurements

Following the completion of the B-mode and A-mode US measurements for the sites, the participants performed exercises to stimulate dumbbell shrugs and dumbbell curls. Immediately after each exercise, the stimulated muscle was measured with B-mode and A-mode US devices at the same sites as in the previous test, following the same procedure.

### 2.3. Data Analysis

**Figure 2:**
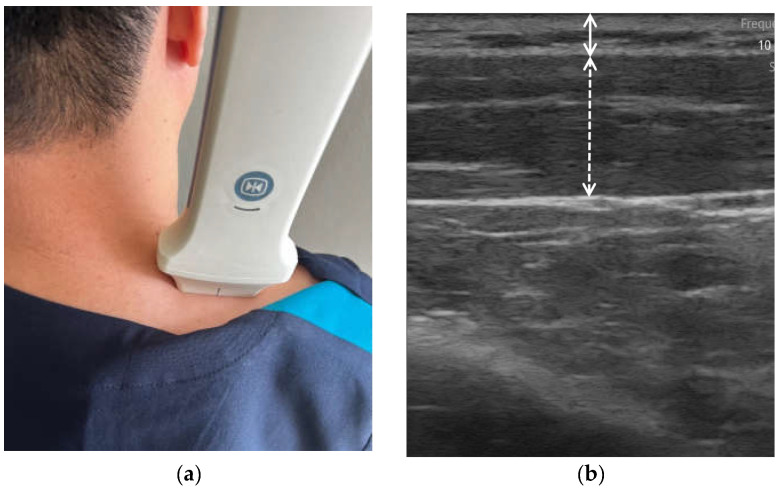
(**a**) B-mode measurement and (**b**) B-mode image analyzed by clinical expert (fat layer for straight line; muscle layer for dash line).

**Figure 3:**
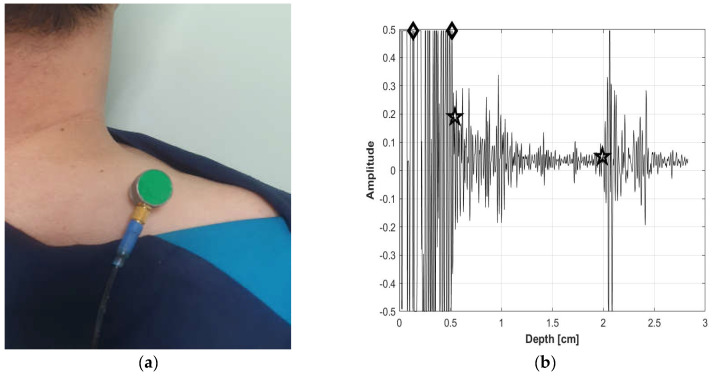
(**a**) A-mode measurement and (**b**) A-mode analyzed signal (fat layer for diamond-marked section; and muscle layer for star-marked section).

As shown in Figure 2, the B-mode US images were analyzed by clinical experts to establish three reference datasets that included data related to muscle, fat thickness, and MQ. These categorized B-mode US images served as the foundation for analyzing the A-mode US signals. The analysis of the A-mode US signals involved the removal of unwanted signals, such as noise, beyond the pulse wave signals emitted from the US probe and the output frequency. The variations for each signal within the preprocessed US signals were analyzed, and the sparkle noise generated within the US signals was eliminated. Distinctive features of the signals originating from the starting point of the skin and subcutaneous fat layer, signals at the endpoint of the subcutaneous fat layer, and signals at the beginning and end points of the muscle layer were identified. As illustrated in Figure 3, this differentiation allowed for the classification of subcutaneous fat and muscle layers, enabling the measurement of SFT and MT. For the portion classified as the muscle layer, the fascia surrounding the muscle was removed to facilitate MQ assessment, which was subsequently performed. Signal enhancement techniques, including filtering, normalization, and noise reduction algorithms, were applied to enhance signal quality. A variation analysis method was employed to capture the unique features within each signal and contribute to the accurate identification of tissue boundaries. Furthermore, a specific algorithm was used for sparkle noise removal to ensure precise measurements. The MQ assessment involved quantifying the quality of the muscle tissue within the identified regions.

### 2.4. Exercise

The participants exercised using 3 kg dumbbells during one session, performing at least three sets of 10 repetitions, with additional sets as needed to stimulate the muscles.

#### 2.4.1. Dumbbell Shrugs Targeting Upper Trapezius Muscle

The participants were instructed to stand shoulder-width apart, grab a pair of dumbbells, and hold them on their sides. While engaging their cores and maintaining a neutral head and neck position, they slowly raised their shoulders straight toward their ears. Subsequently, they repeated the movement by slowly lowering their shoulders to the starting position.

#### 2.4.2. Dumbbell Curl Targeting Biceps Brachii Muscle

The participants were instructed to stand shoulder-width apart and hold dumbbells on their sides with their palms facing forward. While engaging their core and keeping their arms at length with a slight bend in their elbows, they slowly bent their elbows until their lower arms made contact with their upper arms. They then slowly straightened their elbows to return to their starting position.

### 2.5. Statistical Analysis

The data analyst refrained from conducting US measurements before proceeding with the statistical analysis. SPSS (version 21.0; SPSS Inc., Chicago, IL, USA) was used for the statistical analysis. For all tests, *p* < 0.05 was considered statistically significant. Initially, the demographic characteristics of the sample were outlined based on sex. The statistical significance of the differences in measurements between men and women was assessed using Student’s t-test. Validity analyses of the A-mode device were performed using the B-mode measurements. Equivalence tests of means showed that a difference of 1 mm as delta indicated no significant mean differences (MDs) between A-mode and B-mode measurements. An intra-class correlation coefficient (ICC) value of 0.50 was considered low, values of 0.50–0.75 were considered moderate, 0.75 was considered good, and 0.90 was considered excellent. A Bland–Altman analysis was employed, including the average of the A-mode and B-mode measurements plotted against each participant’s error score, along with the 95% limits of agreement (LOA). Finally, paired t-tests were used to examine the pre- and post-exercise measurements.

## 3. Results

### 3.1. Study Population

Thirty volunteers (15 men and 15 women) completed the study. Upon examining the sample’s characteristics, Table 1 shows significant differences in height and weight between men and women. The men were aged 24–31 years (27.5 ± 2.6 years), with heights ranging from 169.8 to 191.4 cm (175.4 ± 5.9 cm), weights ranging from 54.0 to 94.0 kg (74.5 ± 11.0 kg), and a body mass index ranging from 17.8 to 32.6 kg/m^2^ (24.2 ± 3.2 kg/m^2^). The women were aged 22–44 years (30.5 ± 6.1 years), with heights ranging from 150.0 to 173.3 cm (163.3 ± 6.9 cm), weights ranging from 48.0 to 68.0 kg (56.2 ± 6.9 kg), and a body mass index ranging from 17.4 to 26.2 kg/m^2^ (21.1 ± 2.4 kg/m^2^).

### 3.2. Changes in SFT before and after Exercise 

**Figure 4:**
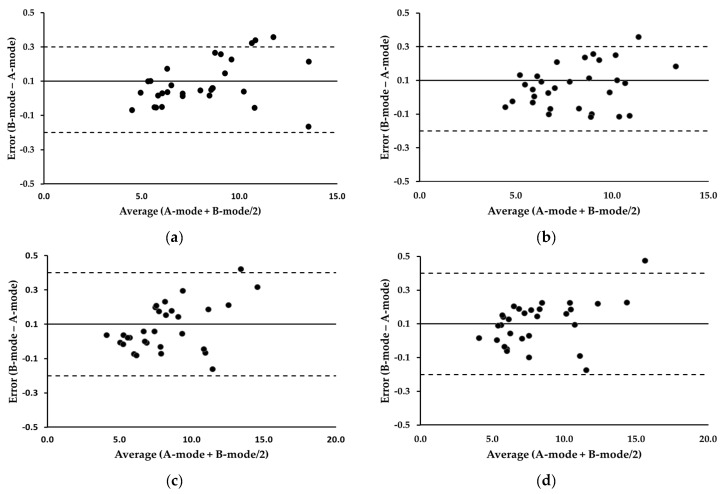
Bland-Altman plots illustrating error scores (B-mode minus A-mode) for the trapezius and biceps brachii. (**a**) Trapezius, pre-exercise; (**b**) trapezius, post-exercise; (**c**) biceps brachii, pre-exercise; (**d**) biceps brachii, post-exercise. The average of the error scores is presented as a solid line. The 95% LOA are presented as dotted lines. All values are expressed in millimeters.

Table 2 summarizes the MD and ICC analyses. The MDs between the two devices consistently remained below 0.15 mm across all the measurements. The equivalence tests of the means indicated no significant differences between the two modes (*p* > 0.05). The ICCs surpassed the excellence threshold of 0.90 for all measures. Bland–Altman plots illustrating the residual scores for the trapezius and biceps brachii are shown in Figure 4. The average of the error scores (B-mode − A-mode) at the trapezius and biceps brachii was 0.1, with 95% LOA ranging from −0.2 to 0.3 and −0.2 to 0.4, respectively. These values did not change pre- and post-exercise in both muscles. The correlation coefficients between the error scores and average scores (A-mode + B-mode/2) at the trapezius were 0.379 pre-exercise and 0.284 post-exercise (*p* > 0.05), indicating no evidence of systematic bias. Similarly, the correlation coefficients at the biceps brachii were 0.472 pre-exercise and 0.421 post-exercise (*p* > 0.05), suggesting that the errors were evenly distributed.

### 3.3. Changes in MT before and after Exercise

**Figure 5:**
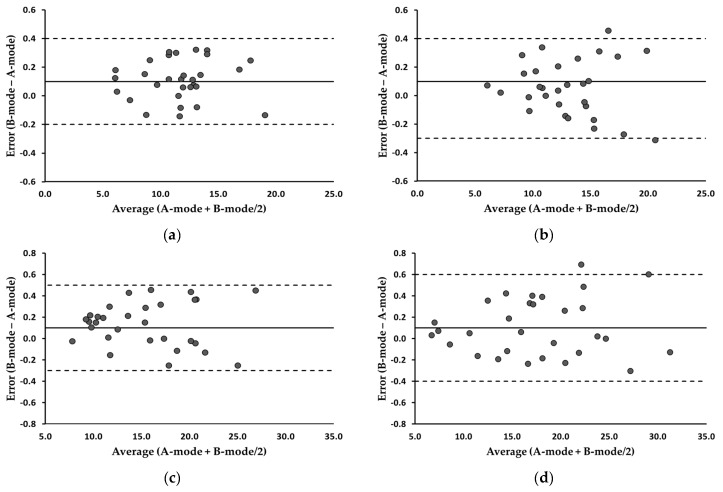
Bland-Altman plots illustrating error scores (B-mode minus A-mode) for the trapezius and biceps brachii. (**a**) Trapezius, pre-exercise; (**b**) trapezius, post-exercise; (**c**) biceps brachii, pre-exercise; (**d**) biceps brachii, post-exercise. The average of the error scores is presented as a solid line. The 95% LOA are presented as dotted lines. All values are expressed in millimeters.

Concerning the MDs and ICCs of MT, the MDs were below 0.22 mm across all the measurements, indicating no significant differences between the A-mode and B-mode (*p* > 0.05). For all measures, the ICCs surpassed 0.99, as shown in Table 3. Figure 5 depicts the Bland–Altman plots illustrating the residual scores for the trapezius and biceps brachii. The average of the error scores (B-mode − A-mode) at the pre- and post-exercise trapezius was the same at 0.1, with 95% LOA ranging from −0.2 to 0.4 and −0.3 to 0.4, respectively. The average of the error scores at the pre- and post-exercise biceps brachii was the same at 0.1, with 95% LOA ranging from −0.3 to 0.5 and −0.4 to 0.6, respectively. The correlation coefficients between the error scores and average scores (A-mode + B-mode/2) at the trapezius and biceps brachii ranged from −0.103 to 0.087 (*p* > 0.05), indicating no evidence of systematic bias.

### 3.4. Changes in MQ before and after Exercise

**Figure 6:**
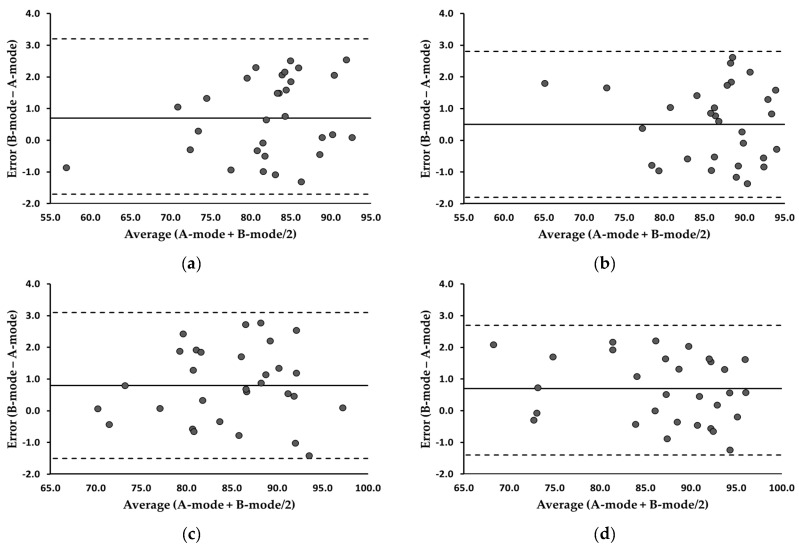
Bland-Altman plots illustrating error scores (B-mode minus A-mode) for the trapezius and biceps brachii. (**a**) Trapezius, pre-exercise; (**b**) trapezius, post-exercise; (**c**) biceps brachii, pre-exercise; (**d**) biceps brachii, post-exercise. The averages of the error scores are presented as solid lines. The 95% LOA are presented as dotted lines.

Table 4 summarizes the MQ analyses. The MDs remained below 1 mm across all the measurements, with the ICCs consistently surpassing 0.95. In the MQ between the two modes, the equivalence tests of means indicated no significant differences (*p* > 0.05), and the ICCs were considered excellent. Examining Figure 6, the Bland–Altman plots revealed that the 95% LOA at the pre- and post-exercise trapezius were −1.7–3.2 and −1.8–2.8, respectively. Additionally, the 95% LOA at the pre- and post-exercise biceps brachii were −1.5–3.1 and −1.4–2.7, respectively. The correlation coefficients between the error scores (B-mode − A-mode) and average scores (A-mode + B-mode/2) at the trapezius and biceps brachii ranged from −0.207 to 0.268 (*p* > 0.05), suggesting no evidence of systematic errors.

### 3.5. MT and MQ Change before and after Exercise in the A-Mode and B-Mode

As shown in Table 5, the study results statistically confirmed a significant increase in the MT and MQ of the trapezius in all the participants after the exercises (*p* < 0.05). The biceps brachii MT significantly increased after the exercises (*p* < 0.05), but this enhancement was not apparent in the MQ of the biceps brachii after the exercises. In the groups, there was no notable increase in the MT and MQ of the trapezius in the women and the biceps brachii in the men. These findings were consistent in both the A- and B-modes.

## 4. Discussion

In this study, to evaluate the accuracy of A-mode US in measuring the SFT, MT, and MQ, we compared the measurements from A-mode US with B-mode US measurements. We introduced exercises to induce various muscle changes and confirmed that A-mode US could detect these post-exercise muscle changes, similar to B-mode US. The comprehensive distribution of muscles throughout the body can offer important diagnostic insights for various myopathies [3,4]. Therefore, we examined specific muscles at different sites. To the best of our knowledge, this is the first study to validate A-mode US as a clinically viable method for assessing MT and MQ, which refer to the degree of adipose and/or connective tissue infiltration into muscles [14].

Recent studies have shown the validity of muscular EI as a marker of MQ [5,9,15]. In a B-mode US image, the EI refers to the tissue’s ability to reflect and absorb US, that is, the brightness of the image obtained through the derived US. The greater the brightness of the image, the higher the proportion of slow-twitch muscle fibers or the presence of intramuscular adipose tissue [5,9]. Therefore, EI reflects the presence of intramuscular adipose tissue and muscle density [5,9]. Additionally, as the EI value increased, MQ and grip strength tended to decrease [5,9,15].

EI is recognized as a noninvasive, readily available, and cost-effective technique compared to other imaging systems [5,9,15,16]. EI is determined by the pixel density in US images and was initially quantified by visually scoring (arbitrarily depicting whether an image is darker or lighter) US images [15]. Currently, many researchers use image-processing software to assess EI by quantifying the pixel intensity in US images [15]. In this study, to quantify muscle composition from B-mode US images, we defined the EI value as the mean pixel intensity within a region of interest in the muscle using grayscale analysis. It is crucial to establish a critical EI value to differentiate between muscle and fat. We set the threshold as the average value observed before and after the exercises to ensure consistency in assessing MQ before and after the exercises.

In this study, we observed excellent similarity between the A-mode and B-mode assessments of SFT and MT, with MDs between the devices of less than 0.22 mm and correlation coefficients exceeding 0.996 for all measures. The MQ data also demonstrated the high validity of the A-mode, with an MD of less than 0.91 mm and correlation coefficients exceeding 0.95, both before and after the exercises. These findings are consistent with those of previous studies that evaluated the accuracy of the A-mode in measuring SFT [11,12].

We measured the upper trapezius and biceps brachii, which are representative muscles of the upper body [4]. Judicious site selection is important for achieving reliable A-mode US measurements. The muscles, such as the biceps and triceps of the upper body, showed clearly visible continuous bands at the adipose tissue borders (dermis/adipose and adipose/muscle-fascia) and could be easily evaluated with a small margin of error in the B-mode US evaluation [17]. Certain sites, where B-mode scans are easily interpreted, showed a strong correlation with A-mode devices [11,17]. Wagner et al.’s study on US measurements of the SFT also reported the highest correlation coefficients in both A-mode and B-mode at the triceps site among various locations (chest, midaxilla, triceps, subscapula, abdomen, anterior supraliac, and thigh) [11]. In contrast, the variability between the two devices is greatest in the abdomen, the site with the greatest fat thickness [11]. In areas with unclear anatomical structures, such as the abdomen, errors may occur during US measurements, and the interpretation of A-mode and B-mode scans may become complicated. In other words, the accuracy of A-mode US measurements varies depending on the anatomical location, underscoring the importance of selecting sites with simple structures for valid measurements.

To investigate the accuracy of the A-mode under diverse muscle conditions, we measured muscle changes before and after the exercises using both A-mode and B-mode devices. The patterns of change in MT and MQ after the exercise remained consistent in both modes. The A-mode US detected alterations in the MT and MQ post-exercise, mirroring the B-mode findings. The results of this study statistically confirmed a significant increase in the MT of the trapezius and biceps brachii in all participants after the exercises (*p* < 0.05). However, there was no significant increase in the MT of the trapezius muscle in the women or the biceps brachii in the men after the exercises. Exercise-induced transient muscle hypertrophy, primarily attributed to the accumulation of fluids such as water and blood, is a well-established phenomenon [18]. However, the absence of temporary hypertrophy in certain cases may be due to insufficient exercise intensity or variations in individual physiological responses to muscle stimulation [18,19]. In particular, the trapezius muscle, which is used less frequently in daily activities than the other muscles, may face challenges in responding to high-intensity exercise.

When examining the MQ changes before and after the exercises, we observed enhanced MQ in the trapezius muscles of all the participants after the exercises; however, this improvement was not evident in the biceps brachii muscles. Neither MT nor MQ showed significant improvements in the trapezius muscles in the women or the biceps brachii muscles in the men after the exercises, suggesting that the intensity of the dumbbell exercises performed on the trapezius muscles in the women and the biceps muscles in the men might not have been sufficient.

This study had several limitations. First, the sample consisted exclusively of the upper trapezius and biceps brachii muscles of young, healthy individuals. The men were aged 24–31 years with a body mass index of 17.8 32.6–32.6 kg/m^2^. The women were aged 22–44 years with a body mass index of 17.4–26.2 kg/m^2^. In individuals with obesity with thick fat layers, the path for the US to reach the muscle tissue is longer, which reduces the resolution of the images. In addition, it is challenging to accurately distinguish between intramuscular adipose tissue and muscle, thus complicating the accurate measurement of MT or MQ [11,17]. There is a lack of research on the validity of A-mode US for measuring SFT in older individuals and those with obesity, and there are no known studies on parameters such as MT or MQ [11,12]. This suggests that the present results may not be universally applicable across diverse demographic groups or unclear anatomical measurement sites, such as the abdominal areas.

Second, in A-mode measurements, the inherent limitations of ultrasonic signals, such as signal attenuation and absorption, can result in signal distortion. This distortion is influenced by the strength and angle of the probe pressing. Additionally, errors in the B-mode ultrasonic measurements may occur depending on the projection angle of the probe. While the muscle size measurements appeared to be minimally affected by the tilt of the US probe (tilted within 6° perpendicular to the muscle), there were significant changes in the EI [20]. Finally, this study did not investigate inter-examiner reliability. Although the investigators in this study had extensive experience in conducting these measurements, it is conceivable that examiners with varying levels of experience might have generated different results.

Despite these limitations, the data from the present study suggest that low-resolution A-mode US provides valid measurements of SFT, MT, and MQ, similar to those obtained through high-resolution B-mode imaging. Furthermore, it detects exercise-induced changes in muscles, similar to B-mode. B-mode ultrasonography with specialized software is becoming increasingly popular for measuring skeletal muscle composition [4,5,6,7,8]. However, high-resolution B-mode US units typically cost over USD 30,000, with an additional expense of USD 4000 for proprietary software and USD 1100 for a 2D training course [11]. This poses a significant cost barrier for independent sonographers seeking to evaluate muscles in field settings or within fitness establishments. A-mode US can be an affordable and portable alternative for muscle assessment, especially at uncomplicated anatomical sites in healthy individuals. This could serve as an acceptable and practical option for individuals who lack access to prioritized B-mode devices.

Finally, future studies should concentrate on conducting additional experiments that incorporate various US parameters to improve the evaluation method and estimation equations for A-mode muscle assessments. The main parameters of muscle US include MT, fascicle length (FL), pennation angle, EI, and cross-sectional area [21,22]. FL refers to the length of the fascicle path running between the superficial and deep aponeuroses [21,22]. This involves identifying complex structures, such as the fascia, from A-mode US signals. Subsequent research showing A-mode US precision in accurately measuring and distinguishing perimuscular structures, such as the fascia, could establish A-mode US as a dependable tool for future muscle assessment. Furthermore, given that the experimental data in this study focused exclusively on healthy participants, it is imperative to complement them with additional experiments, especially targeting older individuals or those with obesity. A comprehensive data analysis from these extensive studies will help develop A-mode as a useful tool for muscle evaluation.

## 5. Conclusions and Outlooks

We assessed the precision of A-mode US for measuring SFT, MT, and MQ by comparing its measurements with B-mode US measurements. The results across three indices indicated a high level of similarity between A-mode and B-mode, with minimal MDs of less than 0.91 mm and intraclass correlation coefficients exceeding 0.95 for all measures. By introducing exercise to induce diverse muscle changes, we validated that A-mode US effectively detected post-exercise alterations in the muscles, similar to B-mode US. Considering the cost of B-mode US as well as the expertise, training, and time needed to interpret B-mode results, the introduction of B-mode is cost-prohibitive for independent sonographers aiming to assess muscles. Particularly at uncomplicated anatomical sites in healthy individuals, A-mode US can serve as a cost-effective and portable alternative for assessing muscles, particularly in situations with limited resources where B-mode US is not available.

The A-mode US measurement device and analysis program developed in this study are expected to find applications not only in the medical field but also in industrial sectors where high-cost equipment may not be feasible. Particularly in healthcare settings, such as fitness centers, these tools can be used to accurately measure and assess the extent and effectiveness of exercises performed before and after workouts in real time, similar to the precise measurements provided by B-mode US imaging devices. In future, specific utilization strategies will be devised in the sports field using A-mode US measurement devices, and the efficiency of the developed equipment will be verified.

## Figures and Tables

**Table 1:** Study population.

Parameter	Men (*n* = 15, Mean ± SD ^1^)	Women (*n* = 15, Mean ± SD)	*p*-Value
Age (years)	27.5 ± 2.6	30.5 ± 6.1	0.092
Height (cm)	175.4 ± 5.9	163.3 ± 6.9	<0.001
Weight (kg)	74.5 ± 11.0	56.2 ± 6.9	<0.001
BMI (kg/m^2^)	24.2 ± 3.2	21.1 ± 2.4	0.006

^1^ SD; standard deviation.

**Table 2:** Changes in SFT pre- and post-exercise.

	Pre-Exercise				Post-Exercise			
Biomarker	A-Mode	B-Mode	MD	ICCs	A-Mode	B-Mode	MD	ICCs
Trapezius	8.06 ± 2.45	8.14 ± 2.50	−0.09	0.998	7.99 ± 2.20	8.06 ± 2.24	−0.06	0.998
Men	7.38 ± 2.08	7.44 ± 2.14	−0.06	0.998	7.40 ± 2.08	7.44 ± 2.11	−0.04	0.998
Women	8.73 ± 2.67	8.84 ± 2.71	−0.11	0.998	8.59 ± 2.23	8.67 ± 2.26	−0.08	0.998
Biceps brachii	8.19 ± 2.58	8.27 ± 2.65	−0.08	0.998	8.07 ± 2.80	8.18 ± 2.86	−0.10	0.998
Men	7.00 ± 1.37	7.05 ± 1.44	−0.05	0.998	6.49 ± 1.03	6.58 ± 1.06	−0.09	0.997
Women	9.31 ± 2.78	9.41 ± 2.85	−0.10	0.998	9.39 ± 3.08	9.53 ± 3.17	−0.14	0.998

**Table 3:** Changes in MT pre- and post-exercise.

	Pre-Exercise				Post-Exercise			
Biomarker	A-Mode	B-Mode	MD	ICCs	A-Mode	B-Mode	MD	ICCs
Trapezius	11.56 ± 3.13	11.68 ± 3.14	−0.11	0.998	13.00 ± 3.49	13.06 ± 3.47	−0.06	0.998
Men	12.70 ± 3.29	12.80 ± 3.28	−0.10	0.998	14.82 ± 3.01	14.80 ± 3.01	0.02	0.998
Women	10.43 ± 2.59	10.56 ± 2.64	−0.12	0.998	11.18 ± 3.00	11.31 ± 3.06	−0.14	0.998
Biceps brachii	15.31 ± 5.04	15.45 ± 5.03	−0.14	0.999	17.46 ± 6.36	17.58 ± 6.39	−0.11	0.999
Men	17.52 ± 5.83	17.57 ± 5.79	−0.06	0.999	20.11 ± 7.62	20.19 ± 7.62	−0.08	0.999
Women	14.28 ± 3.40	14.50 ± 3.49	−0.22	0.997	16.52 ± 3.51	16.64 ± 3.64	−0.12	0.996

**Table 4:** Changes in MQ pre-and post-exercise.

	Pre-Exercise				Post-Exercise			
Biomarker	A-Mode	B-Mode	MD	ICCs	A-Mode	B-Mode	MD	ICCs
Trapezius	81.80 ± 7.10	82.53 ± 7.43	−0.73	0.981	86.01 ± 6.65	86.53 ± 6.51	−0.51	0.981
Men	80.37 ± 8.48	81.01 ± 8.73	−0.64	0.987	87.06 ± 4.57	87.67 ± 4.74	−0.61	0.951
Women	83.23 ± 5.29	84.05 ± 5.75	−0.83	0.966	84.97 ± 8.27	85.39 ± 7.91	−0.42	0.992
Biceps brachii	84.49 ± 6.70	85.30 ± 6.76	−0.81	0.978	86.53 ± 7.87	87.21 ± 7.65	−0.67	0.988
Men	87.60 ± 6.80	88.41 ± 6.33	−0.81	0.981	88.38 ± 9.14	88.99 ± 8.49	−0.61	0.993
Women	82.61 ± 5.37	83.37 ± 5.74	−0.77	0.971	86.79 ± 5.39	87.70 ± 5.33	−0.91	0.971

**Table 5:** MT and MQ change before and after exercise in the A-mode and B-mode.

	MT Change		MQ Change	
	*p*-Value in A-Mode	*p*-Value in B-Mode	*p*-Value in A-Mode	*p*-Value in B-Mode
Trapezius muscle	0.0012	0.0019	0.0056	0.0102
Men	0.0000	0.0002	0.0001	0.0003
Women	0.2885 *	0.2867 *	0.4834 *	0.5868 *
Biceps brachii	0.0231	0.0244	0.1866 *	0.1887 *
Men	0.2675 *	0.2572 *	0.9872 *	0.8795 *
Women	0.0003	0.0008	0.0013	0.0006

*: *p* > 0.05.

## Data Availability

The raw data supporting the conclusion of this article will be made available by the authors without undue reservations.
